# Maximising engagement, motivation and long term change in a Structured Intensive Education Programme in Diabetes for children, young people and their families: Child and Adolescent Structured Competencies Approach to Diabetes Education (CASCADE)

**DOI:** 10.1186/1471-2431-9-57

**Published:** 2009-09-15

**Authors:** Deborah Christie, Vicki Strange, Elizabeth Allen, Sandy Oliver, Ian Chi Kei Wong, Felicity Smith, John Cairns, Rebecca Thompson, Peter Hindmarsh, Simon O'Neill, Christina Bull, Russell Viner, Diana Elbourne

**Affiliations:** 1 Child and Adolescent Psychological Services, University College Hospital, London, UK; 2 Social Sciences Research Unit, Institute of Education, University of London, UK; 3 Department of Practice and Policy, School of Pharmacy, University of London, UK; 4 Centre for Pharmacy Research, School of Pharmacy, University of London, UK; 5 Health Services Research Unit, London School of Hygiene and Tropical Medicine, London, UK; 6 Diabetes Child & Adolescent Services, University College Hospital, London, UK; 7 Department of Biochemistry, Endocrinology, and Metabolism, University College Hospital, London, UK; 8 Diabetes UK, 10 Parkway, London, UK; 9 UCL Institute of Child Health, London, UK; 10 Medical Statistics Unit, London School of Hygiene and Tropical Medicine, London, UK

## Abstract

**Background:**

This trial aims to evaluate effective delivery and cost effectiveness of an innovative structured psycho-educational programme (CASCADE) for young people and their families living with diabetes. The increase in numbers of people being diagnosed with diabetes is posing a challenge for both the UK and the rest of the world. The peak age for diagnosis is between 10 and 14 years of age. There is clear evidence that improved diabetes control from diagnosis in childhood can reduce the incidence and progression of long-term complications. However, despite the development of improved insulin regimens and delivery methods, the overall metabolic control in children and adolescents has improved little in the UK in the past decade. Therefore there is a need for novel interventions and health delivery mechanisms aimed at young people and their families to help improve control and reduce complications, illness burden and costs to the NHS.

**Methods/Design:**

The CASCADE trial is a multi-centre randomised control trial with 26 clinics randomised to control or intervention groups, with 572 children and young people involved in the study. The intervention will be delivered in 4 group sessions, over a 4 month period. A developmentally appropriate curriculum will be delivered to groups of 3 - 4 families, focusing on achievement of increasing competency in self-management of diabetes. The control group will receive standard care from their clinical team, usually consisting of regular 3-monthly clinic visits and telephone contact as required with the clinical nurse specialist and consultant. The primary outcomes of the trial will be change in HbA1c between baseline and 12 months and 24 months post recruitment. Secondary outcomes will include measures related to the economic evaluation, psychosocial outcomes, outcomes related to management of diabetes outcomes, and adherence to the intervention.

**Discussion:**

The trial will be run by independent research and service delivery teams and supervised by a trial steering committee. A data monitoring and ethics committee has been put in place to monitor the trial and recommend stopping/continuation according to a Peto-Haybittle rule. The trial will be conducted according to the principles of MRC Good Clinical Practice (GCP) Guidelines and CTRU Phase III Trial Standard Operating procedures.

**Trial Registration:**

Current Controlled Trials ISRCTN52537669

## Background

### Diabetes burden

The increase in numbers of people being diagnosed with diabetes is posing a challenge for both the UK and the rest of the world. The World Health Organisation has named this a "global pandemic", affecting the lives and welfare of hundreds of millions of people with the condition as well as carers and loved ones. This increase in burden has occurred with type 1 as well as type 2 diabetes. The current estimate of prevalence of Type I diabetes in the UK is 1 per 700-1000 children, yielding a total population aged under 25 of approximately 15,000 -25,000. The peak age for diagnosis is between 10 and 14 years of age.[1]

The long-term complications of diabetes include microvascular and macrovascular disease, reduced life expectancy (on average by 23 years in people with type 1 diabetes) and higher cardiovascular and all-cause mortality.[2] Complications are often first detected in adolescence. There is clear evidence that improved diabetes control from diagnosis in childhood can reduce the incidence and progression of microvascular complications including retinopathy, nephropathy and neuropathy.[3] There is also evidence that later improved control reduces complications, even after a history of poor control. [3-5]

### Failure of new insulins and delivery systems to deliver hoped for benefits

Despite the research evidence of the benefits of intensive insulin regimens and the potential benefits of the introduction of new insulins and methods of insulin delivery (e.g. insulin pumps), overall indices of metabolic control in children and adolescents has improved little in the UK in the past decade; currently only 14-20% of children and young people with type 1 diabetes meet the recommended HbA1c threshold of <7.5%.[6] The reasons for this are unclear; In children and adolescents, failure to improve diabetes control is thought to be related to issues related to self- and family management of the implementation of intensive regimens in daily life and adjustment to diabetes, particularly during the developmental changes of adolescence.[7,8] Given the above, there is a concerted search for novel interventions and health delivery mechanisms to help improve control and reduce complications, illness burden and costs to the NHS. [8-10]

### Educational programmes for modern intensive insulin regimens

The search for improved control has led to intensification of diabetes regimens (by injections as well as pumps) through carbohydrate counting and the use of insulin to carbohydrate ratios for determining preprandial insulin doses, together with the use of correction insulin doses informed by insulin sensitivity ratios. This has led to the development of adult education skills programmes to train patients in the self-management of intensive regimens without substantial professional support - e.g. DAFNE (dose adjustment for normal eating) which have been shown to be effective.[11] and cost-effective.[12] in improving HbA1c by approximately 1% in adults.

### Existing psycho-educational interventions

A recent randomised trial in the US (Svoren et al 2003) showed that an intensive psycho-educational programme allied to case-management resulted in 25% fewer total hypoglycaemic events, 60% fewer severe hypoglycaemic events, and 40% fewer hospitalizations and emergency visits than did standard multi-disciplinary care or simple case management approaches.[13] The importance of integrating medical care and educational and psychosocial interventions has been highlighted in the systematic review published by Hampson et al (1999).[14] The absence of high quality UK-based studies and the need for a programme of primary research on interventions for children and young people was also emphasised.[14]

The review found that the theoretical basis of the majority of completed trials is family therapy. However, there is often very little detail in the papers to be clear which school of family therapy is informing the therapeutic delivery of material. A number of groups have used a family/group based approach and obtained robust effect sizes in either metabolic control and/or psychosocial outcomes. [15-18] The evidence supports the clinical view that developmentally appropriate negotiated responsibility has beneficial outcomes.

Effective components of successful programmes incorporate the integration of medical care and educational and psychological interventions. An example of this is relating self management of blood glucose (SMBG) to other aspects of diabetes to demonstrate how information can be used to guide other management behaviours. Multi-component interventions are more successful than those that focus on only one aspect in improving metabolic control, particularly in adolescents. Additionally, there is evidence that diabetes educators show significant improvements in their skills in educating, supporting and counselling patients when using alternative approaches to traditional didactic methods.[19] Therefore interventions must not only deliver knowledge but ensure it can be put into practice.[20] Caring for children and young people with diabetes is fundamentally different to providing services for adults. It is a complex process that must be firmly focused on the child or young person and their family and/or other carers, supported by the skills and experiences of a wide range of health care professionals. Consideration must be given to the physical and emotional needs of the developing and growing individual along with the social constraints of family, friends, early years and school, as well as adapting to different developmental stages over time. A key component of effective chronic care management involving young people and their families or carers is establishing and maintaining the motivation that will enable them to manage the complex juggling act required to achieve effective management of their condition.[21]

One of the major concerns raised by parents and families is access to quality information and advice in a timely fashion and which is delivered in a consistent and evidence based manner in a form that can be easily comprehended by the user. Conventional expert-centred approaches relying on professional advice are usually unavailable out of hours, which may be one reason for the relatively low uptake of intensive management of type 1 diabetes in the UK. Intensive self-management programmes potentially offer a cost-effective way of meeting family needs while also overcoming professional resource limitations.

The UCL Hospitals diabetes team in partnership with families has developed an innovative psycho-educational programme (CASCADE) that promotes engagement, motivation and flexible self management, delivered in a clinic setting with promising improvements in self management, psychological adjustment and long term metabolic control. Previous work that has used psychological approaches to promote engagement, motivation and behaviour change, which resulted in a significant reduction in HbA1c,.[21] have been incorporated into the psycho-educational programme. The intervention CASCADE is based upon the findings of the HTA review.[14] and our Phase 1 pilot work and includes a number of elements shown to be important in predicting success in improving long-term diabetic control as well as simply transferring knowledge. Small improvements in diabetes control can lead to significant improvements in long term health as well as large savings in health care expenditure. In light of this knowledge, the aim of the trial is to test the hypothesis that CASCADE will provide a clinically effective intervention that could be adopted at low cost throughout the UK, without extensive employment of additional professionals. The Diabetes Control and Complications Trial showed that a 1.5% reduction in HbA1c is associated with a 40% or greater reduction in microvascular complications later in life.[3] There is evidence that reducing the current UK paediatric mean HbA1c by 1% would reduce by up to 50% the risk of developing retinopathy and renal impairment over a 10 year period.[3] Evaluation of the process of implementation, effectiveness and costs effectiveness of CASCADE is being carried out by a multidisciplinary team of researchers from the Social Science Research Unit at the Institute of Education, Medical Statistics Unit and Health Services Research Unit at the London School of Hygiene and Tropical Medicine, The School of Pharmacy and Diabetes UK.

### Objectives

1) To examine the implementation and assess the feasibility of the proposed structured intensive educational programme (CASCADE) provided within a standard clinic setting for a diverse range of young people.

2) To investigate the effects of the above intervention on long term metabolic control of diabetes.

3) To evaluate the impact of the intervention on diabetes-specific quality of life and psychosocial functioning.

4) To investigate the cost effectiveness of the intervention.

## Methods/Design and Discussion

### Design

The trial is a multi-centre cluster randomised control trial involving 572 children and young people from 26 clinics in central and southern England, with integral process and economic evaluation. The process evaluation will monitor the implementation (including extent of uptake) of the intervention in experimental clinics and any alternative intervention (standard care) received by young people and families attending control clinics; document factors influencing the implementation of the intervention; identify components of the intervention which contribute to its effectiveness; assess the acceptability of the interventions to young people, parents and clinic staff; and examine perceived impact of the intervention on outcomes Economic evaluation will include within-trial cost-effectiveness analysis and a cost-utility analysis based on a model combining data from the trial with data from the literature. The perspective adopted will be that of the NHS, that is, the economic evaluation will only consider costs to the NHS and health benefits to patients

### Endpoints of the study

Primary outcomes: Change in HbA1c between baseline and 12 months and 24 months post recruitment.

Secondary outcomes will measure

#### (a) Diabetes outcomes directly related to patient management

(i) Diabetes regimen (insulin delivery/number of injections/insulin types) (ii) Hypoglycaemic episodes (frequency, severity) (iii) Admissions to hospital and reason (e.g. episodes of ketoacidosis, hypoglycaemia and (iv) changes in levels of complexity and competency in self management.[22]

#### (b) Psychosocial outcomes

**(i) **Health related quality of life (QOL): PedsQL with Diabetes module.[23] (ii) Diabetes Family Responsibility Questionnaire.[24] (iii) Strengths and Difficulties Questionnaire (parent report).[25]

#### (c) Diabetes outcomes indirectly related to patient management

(i) Compliance with intervention/control (attendance at intervention sessions) (ii) Service utilisation rate (Clinic attendance/Number of contacts with diabetes nurse specialists and diabetes teams)

Economic evaluation will include within-trial cost-effectiveness analysis and a cost-utility analysis based on a model combining data from the trial with data from the literature. The perspective adopted will be that of the NHS, that is, the economic evaluation will only consider costs to the NHS and health benefits to patients.

It should be noted that it will not be possible to directly evaluate the effects of the intervention on diabetes complications because of low incidence during adolescence.

### Type of study

**Figure 1:**
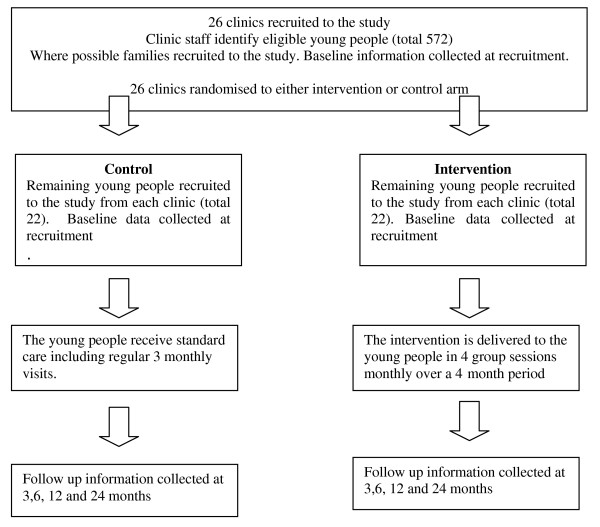
**Flow Chart showing intervention and control treatment**.

The trial is a multi-centre cluster randomised control trial. 572 children and families from 26 clinics in the regional diabetes network covering central and southern England will be recruited. This is an unblinded study as, once recruited and randomised, clinics will be aware of whether or not they are receiving the intervention or control treatment (see Figure 1).

### Randomisation

As this is a cluster randomised trial, clinics will be randomised to intervention or control groups. Randomisation will be minimised by factors likely to influence clinic mean HbA1c; e.g. clinic age (paediatric or adolescent) and the degree of clinic specialisation (district general hospital clinic or teaching hospital/tertiary clinic).

### Interventions - intervention group

The CASCADE intervention is delivered in 4 group sessions (modules), delivered monthly over a 4 month period. Content of each of the four modules is outlined in Table 1. Each module will include the following educational and motivational structure:

**Table 1:** CASCADE module content

**Module 1:** **Living with Diabetes-challenges and choices**	**Module 3:** **Adjusting insulin - pros and cons**
Identification of strengths, resources and abilities Why we need food - a healthy balance How insulin works - happy and unhappy cells What are carbohydrate foods and their effects Insulin regimens and their action profiles Enhancing motivation to change (Post module quiz)	When to adjust insulin When to get help (urgent and non-urgent) Recognising Hyperglycaemia When & how to treat hyperglycaemia Insulin stacking Using an insulin sensitivity ratio Adjusting insulin to food - CHO counting Enhancing motivation to change (Post module quiz)

**Module 2:** **BG testing - pros and cons**	**Module 4:** **Food, activity and exercise**

Reasons for testing BG levels HbA1c and complications Factors that influence the BG level (up and down) Recognising Hypoglycaemia How to treat Hypoglycaemia Enhancing motivation to change (Post module quiz)	Relationship between insulin action & exercise Blueprint for success

i. Solution focused review and goal setting: Trainers invite families to identify recent aspects of diabetic management that have gone well to encourage them to see themselves as experts in their diabetes and to help them identify strengths, resources and abilities. Participants are encouraged to identify what they would find useful or helpful to discuss and think about during the session. This ensures that participants collaborate in the identification of learning objectives

ii. Enhancing motivation to change (Addressing ambivalence): Desired behaviour change choices are discussed using motivational enhancement techniques that address ambivalence. Participants identify the pros and cons of behaviour change acknowledging that any change in behaviour will have both costs and benefits. Motivational interviewing focuses on encouraging the individual to identify the personal and systemic benefits of the desired behaviour to them and their family.

Pre and post session knowledge will be evaluated with specific goals set for what actions the young person plans to initiate in between sessions.

### Interventions - control group

Control families will continue to receive the usual standard care.[26] from their NHS clinical team, this usually consists of regular 3-monthly medical clinic visits plus telephone contact as required with the clinical nurse specialist and consultant. The process evaluation will monitor and describe standard care as well as the CASCADE intervention.

### Duration

The intervention will be delivered over a period of 4 months, and families will be followed-up 12 and 24 months post recruitment.

### Enrolment - inclusion criteria

Participating clinics will be recruited from central and southern England. Participants aged between 8 and 16 years with a diagnosis of type 1 diabetes for one year or more, and HbA1c = 8.5 (defined as mean 12 month HbA1c = 8.5%) will be recruited from within these clinics. An average of 80 - 100 young people per clinic will be eligible to take part in the study with an expected recruitment rate of 25% based on our pilot study.

### Enrolment - exclusion criteria

Table 2 shows the enrolment exclusion criteria.

**Table 2:** Exclusion criteria

**1.**	Participants with significant mental health problems unrelated to diabetes that require specific mental health treatment.
	
**2.**	Participants with significant other chronic illness in addition to diabetes that may be confound the results of the intervention.
	
**3.**	Participants with significant learning disability or lack of command of English sufficient to render them unable to participant effectively in the planned intervention. Note that there is research evidence that the great majority of eligible young people from black or minority ethnic groups in this population have good command of English, although their parents may not. Given the wide range of ethnicities in the sample population, and given the importance of group dynamics to the intervention, it will not be possible to use interpreters to enable parents with poor English to participate. We will ensure that the validity of the study is maintained by including young people with good command of English. They will be eligible to attend sessions by themselves, or with another relative who is one of the primary diabetes carers (such as a sibling, aunt or uncle) who has good command of English and can participate instead of the parents.
	
**4.**	While inclusive elsewhere, we will not recruit clinics which are unlikely to be able to recruit sufficient participants with a good command of English. We anticipate that this pertains to only one clinic within our sample area.
	
**5.**	Young people who have participated in diabetes treatment trials in the 12 months prior to collection of baseline data will not be eligible for this trial.

### Enrolment - Sources/methods of enlisting participants in trial

Participating clinics will be randomly allocated to either the intervention or control arm of the study. To reduce the likelihood of bias resulting from differential recruitment in the two trial arms, clinic staff will be asked to identify eligible young people before the clinics have been randomised, so as to be blind to the random allocation. Eligible families will be sent a letter inviting them to take part in a trial comparing different ways of helping young people to manage their diabetes, making clear that all approaches have potential advantages and disadvantages. Researchers will then visit clinics to meet eligible families, answer any questions that they might have and, if they wish to participate, recruit them to the study. When researchers cannot visit a clinic to meet an eligible family, then recruitment will be carried out by local clinicians using standardised procedures. Timing of clinic appointments (on average every 3 months) means that some eligible families in all clinics will be recruited after randomisation has been carried out. To reduce bias resulting from the lack of blinding at recruitment, families will not be informed of the arm to which their clinic has been randomised until they have decided whether or not to participate in the study.

### Enrolment - Losses to follow-up

We anticipate a drop out rate of approximately 10% and have inflated our sample accordingly (see sample size calculation below). We have been conservative in our estimates of recruitment (25% of eligible), in line with our previous experience. Note also that the motivational elements of this intervention are specifically designed to enhance retention; Drop out during our previous motivational/solution-focused intervention.[21] was minimal (approximately 5%) over a similar number of sessions. For standard care, we estimate that loss to follow-up in our clinic is <10% per year. We will use regional and national diabetes networks and Diabetes UK to maintain contact with those lost to follow-up due to moving.

### Consent

Consent to clinic randomisation will be obtained from the responsible clinician. After clinic randomisation, informed written consent or assent will be obtained for participation in intervention or research procedures from the young person and one parent after clinic randomisation, as per standard procedures for cluster randomised trials.[27] The right of a child or parent to refuse participation without giving reasons will be respected. The child/young person or their parent will remain free to withdraw at any time from the study without giving reasons and without prejudicing further treatment. If a participant withdraws consent from further trial participation their data will remain on file and will be included in the final study analysis. If a participant withdraws consent for their data to be used, the data will be destroyed immediately.

### Sample size calculation

The standard deviation (SD) of HbA1c in the target population is approximately 1.5%. We propose to recruit sufficient young people to allow the trial to detect a difference between groups of 0.5 standard deviations, i.e. 0.75%, with 90% power at a significance level of 0.05 (2-tailed); this is considered to represent a moderate size of effect. This is a highly clinically meaningful effect, given that the Diabetes Control and Complications Trial showed conclusively that a reduction of approximately 1.5% in HbA1c reduced the risk of microvascular complications in adults by 40% (microalbuminuria) to 75% (retinopathy) over 6.5 years,.[3] with similar benefits seen in adolescents.[28] There is considerable other evidence that smaller reductions in HbA1c, of the order of 0.5% are clinically highly significant, e.g. the DCCT found a continuous association of higher HbA1c and greater microvascular risk.[3] The DAFNE trial in adults had an effect size of approximately 1% reduction in HbA1c.[11] Additionally, a reduction of 0.5 SD is half the effect size identified in our pilot work (1.0 SD). Our power calculations are based upon an intracluster correlation coefficient (ICC: the variability in outcome between clinics divided by the sum of the within-cluster and between-cluster variabilities) of 0.1. In the absence of reliable data on which to calculate an ICC for the clinic population, this has been chosen to be compatible with ICCs calculated from the Northern Irish.[29] and Scottish audits,.[30] and databases of ICCs such as . With these assumptions, 13 clinics in each arm with an average of 20 young people in each would be required to detect a difference of half a standard deviation (0.75) with over 90% statistical power at 5% significance. Given the possible loss to follow up of approximately 10%, the target recruitment will be inflated to 22 young people from each clinic. This is in line with the expected 25% anticipated recruitment rate as experienced in our previous intervention study.

## Methods

### Data collection

#### A. HbA1c

This will be collected in two ways because of problems with standardisation.

1. Venepuncture at baseline and 12 months and 24 months post recruitment: For the primary outcome, HbA1c will be collected by venepuncture (by a skilled local clinician) and transported to a single laboratory (University College Hospital) for analysis. This will ensure direct comparability of results from all clinics.

2. Routine clinical measurement of HbA1c (secondary outcomes): It is standard practice to measure HbA1c 3 monthly and the researchers will assist the clinic staff in ensuring that these data are obtained and recorded for all participants at 3 and 6 months post recruitment. Values from the different clinics will be standardised to DCCT aligned values using an accepted approach.

#### B. Questionnaires

A range of diabetic, economic, psychosocial and quality of life data will be collected at baseline and 12 and 24 months post recruitment using standardised questionnaires. Participants, parents and clinic staff as appropriate will complete questionnaires in clinic. Researchers will provide support where necessary.

#### C. Economic data

In addition to HbA1c and questionnaire data on service use, data on costs of the intervention will be carefully recorded by researchers and clinic staff.

#### D. Process evaluation data

Qualitative and quantitative data will be collected through i) observation of training of clinic staff and delivery of intervention sessions with young people and parents ii) focus groups with young people and parents iii) interviews with trainers, clinic staff delivering sessions to young people and other stakeholders iv) structured questionnaires with young people and clinic staff. Trained researchers who have extensive experience of working with young people and other staff and who have no involvement in the design or delivery of the intervention will carry out all data collection.

### Proposed type and frequency of data analysis

#### Quantitative data analysis

HbA1c data and other quantitative data from structured questionnaires will be analysed using STATA (StataCorp, Statistical Software: Release 10.0. 2005, Stata Corporation: College Station, TX.). All primary analyses will be carried out according to the principle of intention-to-treat and taking into account the clustering. Every effort will be made to obtain outcome measures on participants, even if some drop out during the course of the group sessions. The data will be analysed by multiple regression modelling, fitting baseline measures of outcomes as covariates. The small groups in which experimental and control interventions are delivered will be fitted as a random effect. A small number of secondary analyses based on explicit hypotheses, such as subgroup and explanatory analyses considering compliance with the interventions, will be specified in advance. Interim analyses will be reported in confidence to the independent Data Monitoring Committee.

#### Qualitative data analysis

Data from observations, interviews and focus groups as well as open-ended questionnaire responses will be analysed using a system of coding and memoing developed by Lofland and Lofland (1995),.[31] facilitated by the use of NVivo software. First, the key topics and issues that emerged from the data will be identified through familiarisation with transcripts or documents. Pertinent excerpts that illustrate emerging themes will be coded and memos written to summarise and synthesise these emerging themes. In an iterative process, researchers will refine their analysis, ensuring that the themes built up are cross-checked with other data. To maximise the validity of our findings, two researchers will separately analyse a sample of the qualitative data and then meet to compare their analyses and agree a framework for full analysis of all data.

#### Analysis for Economic evaluation

The economic evaluation will assess the potential cost-effectiveness of the structured intervention. Cost will be compared to standard education during clinic appointments. A successful intervention will result in better glycaemic control, which could improve life expectancy and improve health-related quality of life (partly as a result of a reduction in complications). The impact on resource use will have two main elements: the cost of delivering the intervention (including the costs of training of relevant staff); and future changes in resource use as a consequence of a successful intervention.

The within-trial analysis will be in the form of a cost-effectiveness analysis estimating the incremental cost per unit change in glycosylated haemoglobin levels achieved 24 months post recruitment. However, such a measure of cost-effectiveness is of limited value in informing decision making because it is not clear how much the NHS should be willing-to-pay in order to achieve improvements in metabolic control, it only permits comparison with a very narrow range of other interventions, and it ignores longer term health benefits and health care cost savings as a result of a successful intervention. Therefore, the cost-effectiveness of the intervention will also be estimated over a longer time horizon and in terms of cost per quality-adjusted life-year gained. This will be achieved by combining information from the trial (the impact on HbA1c and the incremental cost of the structured intensive intervention) with estimates of other parameters from the literature. This will involve constructing a model of future health care resource use and health outcome (in terms of survival and health-related quality of life) based on the changes in HbA1c observed over follow-up. Successful modelling beyond the two year follow-up of the trial will depend particularly on the specification of the relationship between changes in HbA1c and the risk of complications, and on the evidence in the literature on the health utilities associated with different diabetes-related health states. Deterministic and probabilistic sensitivity analyses will be undertaken to reflect the considerable uncertainty regarding these and other aspects of the model.

### Confidentiality

All information collected during the course of the trial will be kept strictly confidential. Information will be held securely on paper and electronically in the SSRU (process evaluation) and LSHTM (outcome evaluation). The study will comply with all aspects of the 1998 Data Protection Act and operationally this will include

1. Consent from children and parents to record personal details including name, date of birth, address and telephone number, GP name and address and NHS number

2. Appropriate storage, restricted access and disposal arrangements for personal and clinical details

3. Consent from children for the data collected for the trial to be used to develop new research. The child's name, address and telephone number will be collected when they are randomised into the trial but all other data collection forms that are transferred to or from the CTRU will be coded with a trial number and will include two identifiers, usually their initials and date of birth.

4. Consent from patients for access to their medical records by responsible individuals from the research staff, where it is relevant to trial participation

### Ethical Approval

The trial will be performed in accordance with the recommendations guiding physicians in biomedical research involving human participants adopted by the 18th World Medical Assembly, Helsinki, Finland, 1964, amended at the 59th World Medical Association General Assembly, Seoul, October 2008. The study has been approved by the UCL/UCLH Research Ethics Committee (REC; reference number 07/HO714/112).

### Organisation and Governance

#### Project Management Group (PMG)

A project management group will be established and will be responsible for the day to day management of the trial. The group will comprise the principal investigators and project staff from each of the institutions involved in the trial. The group will meet monthly in person and by telephone.

The study combines expertise from five multidisciplinary institutions with strong cross professional links:

• Development and implementation of the structured psycho-educational intervention will be carried out by the UCLH diabetes team, led by the principal investigator Dr Deborah Christie.

• Coordination of the trial including recruitment of trials participants, ensuring procedures for obtaining informed consent from participants, data collection and analysis of process data will be carried out by a team of independent researchers from the Social Science Research Unit at the Institute of Education and UCL School of Pharmacy. Trial coordination will be led by Dr Vicki Strange

• A team from the Medical Statistics Unit (led by Prof Diana Elbourne and Dr Elizabeth Allen) at the London School of Hygiene and Tropical Medicine will provide expertise regarding trial design and undertake the randomisation and all statistical analysis.

• Professor John Cairns, a member of the London School of Hygiene and Tropical Medicine Health Services Research Unit, will supervise all economic aspects of this research

### Trial steering committee

The Steering Committee will approve the main study protocol, monitor and supervise the trial towards its interim and overall objectives, review relevant information from other sources, consider the recommendations of the DMC, and resolve problems brought by the trial co-ordinating centres. The committee will comprise an independent chairperson and 2 independent members, as well as the members of the project management group. This represents all the different disciplines involved in the trial.

#### Data Monitoring Committee

An independent Data Monitoring Committee (DMC) will review, in strict confidence, data from the trial approximately half way through the recruitment period. The Chair of the DMC may also request additional meetings/analyses. In the light of these data, and other evidence from relevant studies, the DMC will inform the Steering Committee, if in their view i) there is proof beyond reasonable doubt that the data indicate that any part of the protocol under investigation is either clearly indicated or contra-indicated, either for all patients or for a particular subgroup, or ii) it is evident that no clear outcome will be obtained with the current trial design.

Unless modification or cessation of the protocol is recommended by the DMC, the Steering Committee, collaborators and administrative staff (except those who supply the confidential information) will remain ignorant of the results of the interim analysis.

#### Membership of the Data Monitoring Committee

Professor Christopher Kelnar, University of Edinburgh; Dr Robert Coe, Durham University; Dr Chris Patterson, Queen's University Belfast; Dr Darren Ashcroft, School of Pharmacy and Pharmaceutical Sciences.

### Publication policy

The results of the study will conform to the CONSORT statement for the reporting of cluster RCTs.[32] and be disseminated widely to the clinical community as well as to participants. To safeguard the scientific integrity of the trial, data from this study will not be presented in public or submitted for publication without requesting comments and receiving agreement from the Trial Steering Committee. As the success of the trial depends on the collaboration of many people, the primary results of the trial will be published by the group as a whole although the paper will be written by a smaller writing committee, and a table of contributors will delineate individual investigators' personal contributions to the study.

### Advisory group of research and service users

Ensuring that service users and potential users of research are involved in the design and governance of the proposed trial is a key objective of this study. To facilitate the involvement of users, the research team will convene a steering group of research and service users. This will include representatives from key policy makers and clinicians. This will meet three times during the study and will provide an opportunity for the research team to consult about the research design and methods for data collection, choice of outcomes and methods for data analyses. This advisory group will have an important role in interpreting initial findings and developing dissemination strategies.

## Abbreviations

BG: blood glucose; BM: blood glucose monitoring; CASCADE: Child and Adolescent Structured Competencies Approach to Diabetes Education; CTRU: Trials Research Unit; DAFNE: dose adjustment for normal eating; DCCT: diabetes control and complications trial; DKA: diabetic ketoacidosis; DMEC: Monitoring and Ethics Committee; GCP: good clinical practice; HbA1c: glycosylated haemoglobin; HTA: Technology Assessment; ICC: intracluster correlation coefficient; MRC: Medical Research Council; NHS: National Health Service; PedsQL: Paediatric Quality of Life Inventory; QOL: quality of life; RCT: randomised control trial; REC: Research Ethics Committee; SD: standard deviation; SMBG: self management of blood glucose; UK: United Kingdom; UCL: University College London.

## Competing interests

The authors declare that they have no competing interests.

## Authors' contributions

All authors contributed to the design of the study, obtaining funding, the refinement of the protocol for ethical approval, the drafting of the manuscript and the reading and approval of the final version. JC contributed to the design of the economic evaluation. DC and BT contributed to the intervention module development, and the development and implementation of the pilot education programme. CB wrote the initial draft of the manuscript. All authors read and approved the final manuscript.

## Pre-publication history

The pre-publication history for this paper can be accessed here:


